# Risk factors for loss to follow‐up after the start of direct‐acting antiviral treatment for hepatitis C virus infection

**DOI:** 10.1002/jgh3.12855

**Published:** 2022-12-30

**Authors:** Akifumi Kuwano, Masayoshi Yada, Kazuki Kurosaka, Kosuke Tanaka, Akihide Masumoto, Kenta Motomura

**Affiliations:** ^1^ Department of Hepatology Iizuka Hospital Iizuka Japan

**Keywords:** direct‐acting antivirals, glecaprevir and pibrentasvir, hepatitis C virus, history of injection drug use, loss to follow‐up

## Abstract

**Background and Aim:**

Direct‐acting antivirals (DAAs) have recently been developed to treat hepatitis C virus (HCV) infection. Additionally, interferon‐free DAA treatment has improved liver function and reduced the risk of hepatocellular carcinoma (HCC) following HCV eradication. Previous studies on HCV have focused mainly on the treatment rate and the risk of developing HCC, and less attention has been given to loss to follow‐up (LTFU) after DAA treatment. Therefore, the present study aimed to identify the definitive risk factors for LTFU after the start of DAA treatment.

**Methods:**

Between September 2017 and March 2022, 296 patients receiving glecaprevir and pibrentasvir for HCV infection were enrolled in this study. The incidence of LTFU following DAA treatment and the risk factors contributing to LTFU were identified using the patients' clinical characteristics.

**Results:**

In the present study, 75 patients (25.3%) interrupted their follow‐up visits. Multivariate logistic analysis revealed a history of injection drug use (hazard ratio [HR], 1.81; *P* = 0.017), treatment duration (8 weeks) (HR, 3.51; *P* = 0.0033), and age <70 years (HR, 1.9; *P* = 0.0422) as independent factors associated with LTFU after the start of DAA treatment.

**Conclusion:**

Young patients and those with injection drug use are likely to discontinue their follow‐up visits after the start of DAA treatment for HCV infection. Therefore, these patients require strict supervision.

## Graphical abstract

Young patients and those with a history of injection drug use are more likely to discontinue their follow‐up visits after the start of direct‐acting antivirals treatment for hepatitis C virus infection.

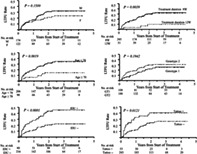

## Introduction

Hepatitis C virus (HCV) has a major impact on public health, infecting approximately 170 million people worldwide and with an estimated global incidence of 3–4 million new infections per year.^1^ HCV infection is a risk factor for liver cirrhosis and hepatocellular carcinoma (HCC).^2^ The HCC risk increases 17‐fold in HCV‐infected patients compared with HCV‐negative patients.^3^

Interferon‐free direct‐acting antivirals (DAAs) were developed in 2014, enabling treatment with high cure rates, shorter duration, and minimal side effects.^4^ Glecaprevir and pibrentasvir (GLE, PIB) are inhibitors of HCV nonstructural protein 3/4A protease and nonstructural protein 5A, respectively.^5^ The combination GLE/PIB has pangenotypic anti‐HCV activity, with a high barrier to resistance, primarily biliary excretion, and negligible renal excretion.^6^, ^7^, ^8^ Therefore, the combination GLE/PIB could be used worldwide for the treatment of HCV‐infected patients, including those with severe renal impairment and those receiving dialysis, or for the retreatment of HCV‐infected patients with previous DAA treatment failure.

Although HCV eradication with DAAs reduces the incidence of HCC, the unexpected early occurrence of HCC after sustained virological response (SVR) has been reported.^9^, ^10^, ^11^, ^12^ The increasing number of patients achieving HCV clearance with DAAs and the continued risk of hepatocarcinogenesis even after SVR requires the follow‐up of these patients. Loss to follow‐up (LTFU), which refers to patients who start care but voluntarily stop it, is a problem for those with chronic disease.^13^, ^14^

Recently, a study group proposed a clarified and slightly extended care continuum (15). The Consensus HCV Cascade of Care is based on the WHO indicators, and it can be divided into four key steps (the four WHO indicators) and three supplementary steps, as follows: (i) estimated HCV prevalence, (ii) diagnosed with chronic HCV, (iii) linked to HCV care, (iv) liver disease assessed, (v) started on treatment in (year), (vi) achieved SVR in (year), and (vii) accessed chronic post‐SVR care.^15^ Most studies stop reporting on the care cascade the moment HCV prevalence and diagnosis is reached. Data on how many patients actually receive surveillance after the start of DAA treatment are lacking. A clear understanding of how patient characteristics influence LTFU can assist follow‐up programs to institute measures for improving patient adherence directed at those with a high risk for interruption of follow‐up.

The present study aimed to identify the incidence rate and risk factors for LTFU after the start of DAA treatment for HCV, in Japan.

## Methods

### Patients

We conducted a single‐center prospective study to determine the incidence rate and risk factors for LTFU after the start of DAA treatment for HCV. A total of 325 patients with HCV infection who received GLE/PIB treatment between September 2017 and March 2022 were included at the Iizuka Hospital, Fukuoka, Japan. We did not obtain sufficient patient information on other DAA treatments except GLE/PIB.

The present study was conducted in accordance with the guidelines of the Declaration of Helsinki, and was approved by the Ethics Committee of Iizuka Hospital (approval no. 17170). Written informed consent was obtained from all patients prior to DAA treatment. Patients who had a history of HCC and died during DAA treatment were excluded; 296 patients were enrolled, and 75 patients (25.3%) were LTFU after the start of treatment.

We explained the importance of HCV eradication and HCC surveillance to all patients before DAA treatment. Specifically, we explained the necessity of visits every 4 weeks during DAA treatment, every 3 months until SVR24 (SVR 24 weeks after treatment), and 3–6 months after SVR24.

We defined LTFU as nonattendance to any appointment with hepatologists after the start of DAA treatment. LTFU did not include a nonscheduled visit before 1 month after the scheduled appointments. Patients who died or who were transferred to a referral hospital were excluded from the definition of LTFU.

We collected the patients' data using medical questionnaires and interviews performed by the attending doctors. The questionnaire was used to collect demographic information and medical history of the patients, with special emphasis on comorbidities and lifestyle.

HCV RNA was extracted from 200 μL of serum using the Qiagen miRNeasy serum‐plasma kit (Qiagen, Hilden, Germany) in accordance with the manufacturer's instructions. Using a commercially available device, a TaqMan polymerase chain reaction method (COBAS AmpliPrep/COBAS TaqMan HCV test version 2 [Roche Molecular Diagnostics, Pleasanton, CA, USA]; lower limit of quantification, 1.6 log 10 IU/mL; lower limit of detection, 1.2 log 10 IU/mL) was used to detect HCV RNA, in accordance with the manufacturer's instructions. HCV genotyping was also performed with the commercial Versant HCV Genotype 2.0 line probe assay (LiPA 2.0; Siemens Healthineers, Erlangen, Germany), and HCV sequencing for detection of HCV genotypes 1a, 1b, 2, 3, 4, and 6 in serum samples was performed in accordance with the manufacturer's instructions.

The fibrosis‐4 (FIB‐4) index was used to evaluate fibrosis clinically before and after DAA treatment. FIB‐4 index was calculated using the following formula: FIB‐4 index = [aspartate aminotransferase (AST; IU/L) × age (years)/platelet count (10^9^/L) × alanine aminotransferase (ALT; IU/L)^1/2^].^16^

We defined SVR as a serum HCV RNA viral load below the lower limit of detection measured at least 12 weeks after the end of treatment.^17^ Liver cirrhosis was diagnosed on the basis of liver histopathology, transient elastography, or the presence of gastroesophageal varices. HCC surveillance was performed by measuring serum α‐fetoprotein levels and imaging examinations, namely ultrasonography, computed tomography, and magnetic resonance imaging, 3–6 months after DAA treatment.^18^

### Statistical analysis

The data are presented as median (range) (Tables 1 and 2). Statistical analyses were performed using the Kaplan–Meier method, log‐rank test, and Cox proportional hazards regression analysis using JMP statistical software (version 11.0 for Windows; SAS Institute, Inc., Cary, NC, USA). All *P*‐values were derived from two‐tailed tests, and *P* < 0.05 was considered statistically significant.

**Table 1:** Baseline characteristics of the patients

*n*	296
Age (years)	61 (51–71)
Age >70 years	90
Male/female	174/122
Treatment history for HCV	46
LC	62
Follow‐up period (days)	653 (324–1128)
Treatment duration	
8 weeks	245
12 weeks	51
IDU	80
Tattoo	53
GT	
1B	108
2A	115
2B	73
GT2	188
HCV‐RNA (log IU/mL)	6.1 (5.3–6.7)
Blood test at SOT	
Plt (×10^4^/mm^3^)	19 (15–24.3)
TB (mg/dL)	0.7 (0.6–0.9)
AST (IU/L)	39 (25–70)
ALT (IU/L)	40 (21–82)
Alb (g/dL)	4.1 (3.8–4.3)
AFP (ng/mL)	3.45 (2.2–5.98)
FIB‐4 index	2.00 (1.25–3.48)
HCC occurrence	5

Data are expressed as median and interquartile range.

AFP, α‐fetoprotein; Alb, albumin; ALT, alanine aminotransferase; AST, aspartate aminotransferase; FIB‐4 index, fibrosis‐4 index; GT, genotype; HCC, hepatocellular carcinoma; HCV, hepatitis C virus; IDU, injection drug use; LC, liver cirrhosis; Plt, platelet count; SOT, start of treatment; TB, total bilirubin.

**Table 2:** Characteristics of the patients with or without loss to follow‐up

	LTFU (−)	LTFU (+)	*P*‐value
*n*	221	75	
Age (years)	63 (53–72.5)	52 (39–64)	<0.0001
Age >70 years	101	11	
Male/female	125/96	49/26	0.2218
Treatment history for HCV			
LC	59	3	<0.0001
Follow‐up period (day)	903 (530–1280.5)	210 (84–438)	<0.0001
Treatment duration			0.0012
8 weeks	174	71	
12 weeks	47	4	
LTFU timing			
SOT–EOT		4	
EOT–SVR12		25	
SVR12–SVR24		10	
SVR24–		36	
IDU	49	33	<0.0001
Tattoo	37	20	0.0266
GT			
1B	99	22	0.0121
2A	98	23	
2B	52	30	
GT2	150	53	0.1303
HCV‐RNA (log IU/mL)	6 (5–6.58)	6.65 (5.83–6.9)	0.0002
Blood test at SOT			
Plt (×10^4^/mm^3^)	18.25 (14.53–24)	21.15 (16.5–26.2)	0.0133
TB (mg/dL)	0.7 (0.6–0.98)	0.7 (0.6–0.9)	0.6613
AST (IU/L)	40.5 (24.25–72.75)	36 (25–67)	0.5794
ALT (IU/L)	37 (21–157.7)	43 (20–76)	0.6562
Alb (g/dL)	4.0 (3.73–4.3)	4.2 (4–4.5)	0.0002
AFP (ng/mL)	3.55 (2.2–6.13)	3.35 (2.28–5.3)	0.4365
FIB‐4 index	2.45 (1.49–4.18)	1.6 (0.95–2.58)	0.0005
HCC occurrence	5	0	
SVR rate			<0.0001
SVR	212	46	
Non‐SVR	4	2	
Not reached	5	0	
Dropout	0	27	

Data are expressed as median and interquartile range.

AFP, α‐fetoprotein; Alb, albumin; ALT, alanine aminotransferase; AST, aspartate aminotransferase; EOT, end of treatment; FIB‐4 index, fibrosis‐4 index; GT, genotype; HCC, hepatocellular carcinoma; HCV, hepatitis C virus; IDU, injection drug use; LC, liver cirrhosis; LTFU, lost to follow‐up; Plt, platelet count; SOT, start of treatment; SVR, sustained virological response; SVR12, SVR 12 weeks after treatment; SVR24, SVR 24 weeks after treatment; TB, total bilirubin.

## Results

### Patient characteristics

The characteristics of the patients enrolled in this study are shown in Table 1. We found an LTFU rate of 25.3%. Table 2 shows the characteristics of the patients not lost to follow‐up (*n* = 221) and lost to follow‐up (*n* = 75) after the start of GLE/PIB treatment. Among the 75 patients lost to follow‐up, 49 were male and 26 were female. The patients' mean age was 52.0 years (range 39–64) and 11 (14.7%) patients were over 70 years of age. Four patients (1.4%) were lost to follow‐up before the expected end of treatment after starting care. Additionally, 25 patients (8.4%) were lost before confirmation of SVR after 12 weeks of treatment (Table 2). The number of genotype 1 and 2 cases was 22 and 53, respectively. Thirty‐three patients (44.0%) had a history of injection drug use (IDU) and 20 patients (26.7%) had tattoos. There were significant differences between patients with and without LTFU for age >70 years (*P* < 0.0001), treatment duration (*P* < 0.0001), IDU (*P* < 0.0001), HCV RNA (*P* = 0.0002), platelet count (*P* = 0.0133), serum albumin (*P* = 0.0002), and FIB‐4 index (*P* = 0.0005). The SVR rate was 98.3% in patents without LTFU and 95.9% in patients with LTFU (*P* < 0.0001). Five patients without LTFU developed HCC during the follow‐up.

### Cumulative LTFU incidence rate

**Figure 1:**
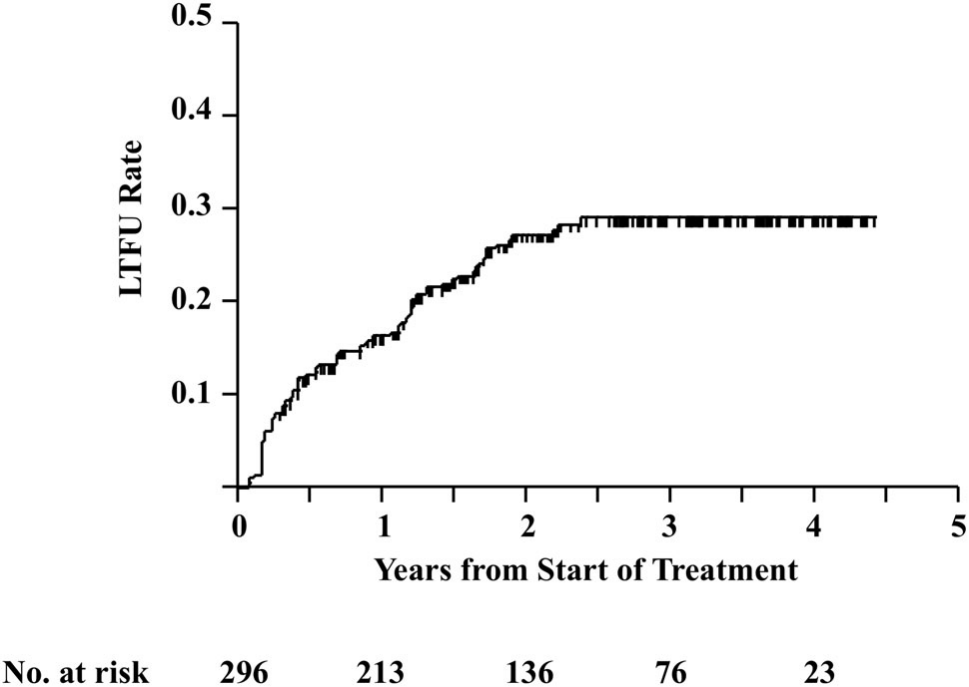
Cumulative loss to follow‐up incidence rate. LTFU, loss to follow‐up; No., number.

The median observation time was 653 days after the start of DAA treatment; 75 patients were LTFU, broken down as follows: 16.1, 27.0, and 28.8% at 1, 2, and 3 years, respectively, by the Kaplan–Meier method (Fig. 1). The number of patients followed up at each time point is presented in Figure 1.

### Risk factors for LTFU after DAA treatment for HCV infection

**Figure 2:**
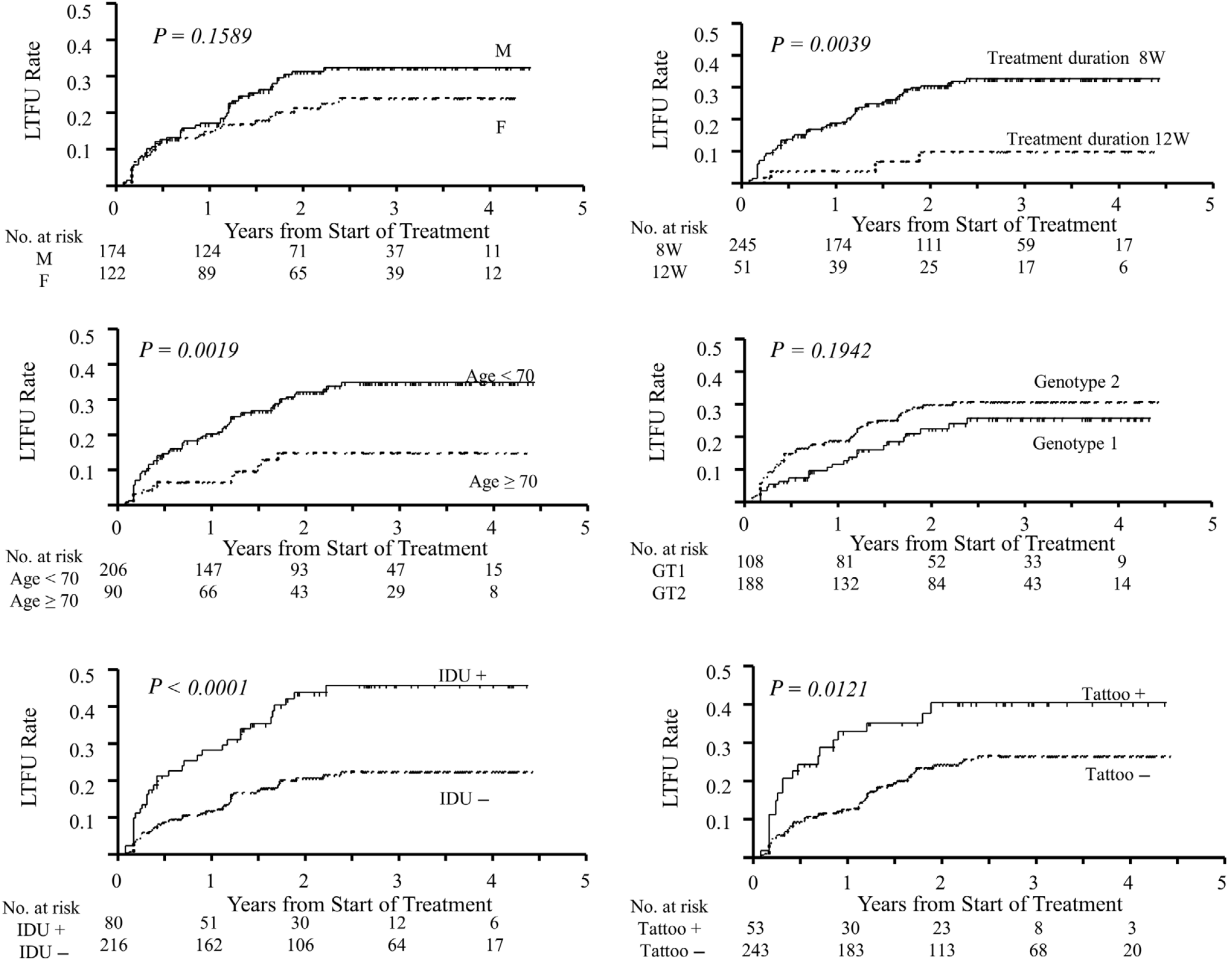
Kaplan–Meier analysis of the cumulative loss to follow‐up (LTFU) rate, stratified on the basis of multiple parameters. F, female; GT, genotype; IDU, injection drug use; LTFU, loss to follow‐up; M, male; No., number.

Figure 2 shows the results of the Kaplan–Meier analysis of the cumulative LTFU rate. There were significant differences between patients with and without LTFU for age (*P* = 0.0019), treatment duration (*P* = 0.0039), IDU (*P* < 0.0001), and the presence of tattoos (*P* = 0.0121).

Table 3 shows the risk factors associated with LTFU after DAA treatment. The potential predictive factors for LTFU were age (<70 years), sex (male), HCV genotype,^2^ treatment duration (8 weeks), IDU, and the presence of tattoos. On the basis of the univariate analysis, age (<70 years), treatment duration (8 weeks), IDU, and the presence of tattoos were identified as risk factors. The results of the multivariate analysis indicated that age (<70 years), treatment duration (8 weeks), and IDU were independent factors significantly associated with LTFU.

**Table 3:** Factors associated with loss to follow‐up

	Univariate			Multivariate		
	HR	95% CI	*P*‐value	HR	95% CI	*P*‐value
Age <70	2.63	1.45–5.29	0.0009	1.9	1.02–3.88	0.0422
Sex: male	1.4	0.88–2.29	0.1561			
HCV genotype 2	1.38	0.86–2.30	0.1892			
Treatment duration: 8 weeks	3.93	1.63–12.91	0.001	3.51	1.45–11.54	0.0033
IDU	2.4	1.51–3.78	0.0003	1.81	1.11–2.92	0.017
Tattoo	1.9	1.11–3.12	0.0199	1.49	0.86–2.50	0.1504

CI, confidence interval; HCV, hepatitis C virus; HR, hazard ratio; IDU, injection drug use.

## Discussion

HCC is currently a major cause of morbidity and mortality in HCV patients who have achieved SVR.^19^, ^20^ Post‐SVR surveillance by liver imaging and α‐fetoprotein measurement every 6 months after SVR is recommended in the cirrhotic population in accordance with international guidelines.^18^, ^21^

A major problem in HCV elimination is LTFU.^22^, ^23^ LTFU inhibits the detection of curable early stage carcinogenesis in patients after DAA treatment. However, there have been few studies evaluating LTFU and non‐adherence to care in the HCV‐infected population. In particular, most data on non‐adherence and patient compliance were analyzed as factors related to treatment efficacy, and patient‐related factors were not considered the primary outcome.^24^, ^25^, ^26^, ^27^

In our study, 75 patients (25.3%) interrupted their follow‐up visits, 4 patients (1.4%) were lost to follow‐up during treatment, and 25 patients (8.4%) left the study before confirmation of SVR 12 months after treatment. Darvishian *et al*. showed that LTFU exceeded viral failure in their real‐world study, impeding the cascade of care. Studies have shown that 0–11% (median 3.4%) of patients are lost to follow‐up during therapy and 0–25% (median 4.9%) after therapy completion, which is similar to the values in our study.^23^

Several reports evaluating LTFU in HCV care have been published recently.^28^, ^29^, ^30^, ^31^ These studies have reported that the factors associated with LTFU are younger age (<45 years),^28^ treatment in hospital,^29^ history of homelessness,^29^, ^30^ mental illness,^30^, ^31^ and IDU.^32^, ^33^ In contrast, the factors associated with retention in care are older age (>60 years) and HIV coinfection.^23^ However, in previous reports, LTFU has been defined as non‐attendance to any appointment in the care cascade at any time, and less attention has been paid to the patients in HCV care after DAA treatment in these reports. Data on how many patients actually receive surveillance after the start of DAA treatment are lacking. This study identified the risk factors for LTFU after the start of DAA treatment as IDU, treatment duration, and age.

The factors that are often associated with LTFU are linked to IDU. Only 36–65% of people with IDU attend follow‐up appointments after the diagnosis of HCV.^34^, ^35^, ^36^ It has been reported that patients with ongoing IDU are susceptible to HCV reinfection.^37^, ^38^ Additionally, treatment duration and age were factors associated with LTFU in this study, which suggested that older patients and those with liver cirrhosis/retreatment might have good adherence to follow‐up plans.

A previous study reported that 30% of patients who received interferon‐based treatment dropped out at 3 years.^39^ In our study, 28.8% patients who received DAA treatment interrupted their follow‐up visits at 3 years. There seemed to be no difference in LFTU rates according to treatment regimen.

Interestingly, the incidence rate of LTFU stopped increasing after almost 2.5 years in this study. This result suggests that LTFU occurs frequently in the early period and that it is necessary to provide early and strong guidance to patients on the importance of judging treatment results and carcinogenesis surveillance to prolong overall survival.^40^

There was no difference in the efficacy of HCV eradication by DAA between patients not lost to follow‐up and those lost to follow‐up in this study. We suggest that aggressive treatment for HCV eradication by DAA is necessary even in the high‐risk LTFU group.

The limitations of this study are its single‐center design, which limited the number of patients, and non‐consideration of the involvement of mental illness in LTFU. In patients with HCV infection, the association between psychiatric disorders and liver failure is common. Patients infected with HCV have a high prevalence (50%) of psychiatric disease.^41^ Depression is the most commonly diagnosed mood disorder in these patients, and it has been extensively associated with barriers to compliance with care and treatment adherence.^42^ In addition, 63.5% of patients were genotype 2 in this study. The prevalence of HCV genotype 1 infection is approximately 70%, while that of HCV genotype 2 is approximately 20–30% in Japan.^43^ The population of this study is different from the general population in Japan.

## Conclusion

In conclusion, young patients and those with IDU are more likely to discontinue their follow‐up visits. Therefore, these patients require strict supervision, and maintaining contact, such as through recalls using available contact information, is important.

## Data Availability

The datasets used and/or analyzed during the current study are available from the corresponding author on reasonable request.
